# Isolation of Microsatellite Loci from the Onion Thrips, *Thrips tabaci*

**DOI:** 10.1673/031.013.3001

**Published:** 2013-04-18

**Authors:** Kazuya Kobayashi, Eisuke Hasegawa

**Affiliations:** Laboratory of Animal Ecology, Department of Ecology and Systematics, Graduate School of Agriculture, Hokkaido University. Sapporo 060-8589, Japan.

**Keywords:** genomic DNA, inbreeding, Menderian inheritance, reproductive mode

## Abstract

*Thrips tabaci* Lindeman (Thysanoptera: Thripidae), a cosmopolitan pest insect, is subdivided into at least three genetic lineages that have different biological characters, such as reproductive mode and resistibility to insecticides. Since the lineages are discriminated only by mitochondrial DNA, there is a possibility of gene flow among lineages at the genomic level. Nine polymorphic microsatellite loci were newly isolated from the taxon. Moderate to high levels of polymorphism were observed, with numbers of alleles ranging from 6 to 12 in 51 individuals. The mean observed and expected heterozygosities ranged from 0.1373 to 0.3725 and 0.5381 to 0.7748, respectively. Contrary to the expectation under Hardy-Weinberg's equilibrium, six of the nine loci exhibited a reduction to homozygosities. However, we confirmed that alleles in all the loci were inherited as Mendeilan characteristics. These new loci will be useful to explore discrimination of lineages and population genetics in this species.

## Introduction

The onion thrips, *Thrips tabaci* Lindeman (Thysanoptera: Thripidae), is subdivided into several lineages based on different biological characters, such as reproductive mode (Toda and Murai 2007), resistance to insecticides (Toda and Morishita 2009), and host preference (Brunner et al. 2004). Although mitochondrial DNA sequences were differentiated among the lineages, no differences in morphology among the lineages were found. Since the mitochondrial DNA shows maternal inheritance, there is a possibility of gene flow among lineages at the genomic level. This study reports nine microsatellite markers that will be useful to examine the existence of gene flows among lineages in *T. tabaci.*

## Materials and Methods

*T. tabaci* was sampled in August 2011 from an onion field in Nanporo town, Hokkaido prefecture, Japan. Genomic DNA was isolated from adult females individually using the DNeasy tissue Kit (Qiagen, www.qiagen.com). Isolation of microsatellite loci was performed following a microsatellite enrichment method (Hamaguchi et al. 2007) using the magnetic beads coated by streptavidine (Promega, www.promega.com). Digested DNA by Sau3AI was ligated to an adapter (Cassete Sau3AI, TaKaRa, www.takara-bio.com) with T4 DNA ligase (TOYOBO, www.toyobo-global.com) and amplified on a 2720 Thermal Cycler (Applied Biosystems, www.appliedbiosystems.com). From the amplified DNA, pieces including microsatellite regions were isolated using a biotinylated oligonucleotide repeats (GT)10 and magnetic beads (Promega). Fragments were cloned using pGEM-T vector (Promega) and the XL1-Blue MRF′ bacterial host strain (Stratagene, www.genomics.agilent.com). Inserted DNA of color-positive clones were amplified by PCR and screened for microsatellite regions by a membrane based “dot-blot” using the detection kit for the biotinylated probe (Imaging-High-Color, TOYOBO). Positive clones were checked for insert size by PCR using an MJ research PTC-100. The 25 µl reaction contained 1 µl template DNA, 0.2 U of Ex-taq (TaKaRa), 0.8 µl each of primer, 2.5 µl of 10x PCR buffer (TaKaRa), and 200 µM of each dNTP. Clones that exhibited a single band of 400–1000 bp were purified using the DNA purification kit (Qiagen) and were sequenced using a DTCS quick start kit (Beckman-Coulter, www.beckmancoulter.com). Sequences were electrophoresed on a CEQ-8000 genetic analyzer (Beckman-Coulter).

Forty-eight inserts contained a microsatellite region with more than 10 repeat units, for which primers were designed. A DNA mix of 30 *T. tabaci* individuals was amplified for each locus, and the products were separated by a polyacrilamide gel electrophoresis (Imai and Hasegawa 2004). Only loci showing multiple bands in this preliminary analysis were checked for allelic polymorphism using dye-labeled primers with Beckman dyes. The PCR cycle consisted of 2 minutes at 96° C, followed by 35 cycles of denaturation for 5 seconds at 98° C, an annealing for 30 seconds at a temperature specific for each primer pair (see Table 1) and an extension of 1 minute at 68° C, and one step of 1 minute at 68° C to complete extension at the end. Each of the 10 µl reaction mixtures contained 2 pmol of each primer, 5 µl of 2 × Mighty Amp Buffer, 0.1 µl of Mighty Amp DNA Polymerase (TaKaRa; 1.25 U/µl), and 1 µl template DNA. The PCR products were electrophoresed with 0.5 µl of size standard 400 (Beckman-Coulter) on a CEQ-8000 genetic analyzer (Beckman-Coulter).

To confirm Mendelian inheritance of alleles on the isolated microsatellite loci, 11 females sampled from the onion field and their offspring produced in the laboratory were genotyped at all the nine loci. A binomial test was conducted to evaluate Mendeilan characteristics of the nine microsatellite alleles. For each locus in which heterozygosity was observed in a mother, whether or not each allele of the mother was observed at equal probability in her offspring was tested. To detect the alleles that offspring inherited from their mother, MATESOFT version 1.0 (Moilanen et al. 2004) was utilized. A genotype of each mother's mate was estimated by subtracting the allele of the mother from the genotype of her offspring. When offspring had the same heterozygous genotype their mother, the genotypes of her mates were estimated from the genotypes of other offspring, so as to explain it by minimum number of mates. And then, the alleles inherited from their mother were detected by excluding the paternal allele from the data of offspring alleles.

## Results and Discussion

Nine loci were found to be polymorphic, and the number of alleles per locus ranged from 6 to 12 in 51 individuals (Table 1). Observed and expected heterozygosities ranged from 0.1373 to 0.3725 and 0.5381 to 0.7748, respectively. Six loci showed significant reductions in heterozygosity from Hardy-Weinberg equilibrium, and linkage disequilibrium was found between TMS58 and TMS59. However, the binomial test to confirm Mendelian inheritance of alleles at the loci showed no significant differences from Mendelian expectation in any loci (Table 2). This result indicates that these alleles are inherited as Mendelian characteristics at all loci. Excess of homozygosities in wild populations might be a result of inbreeding. Further studies will clarify an existence of inbreeding in this species.

The highly polymorphic microsatellite loci described in this study will be useful to study some interesting characters in *T. tabaci.* For example, both sexual and asexual reproduction are known in *T. tabaci* (Jenser and Szénási 2004). Half inheritance of two alleles by meiosis occurs in sexual reproduction and allows generations of genetic diversity. However, Williams (1975) has proposed that there is also a cost of meiosis in sexual reproduction that does not occur in asexual individuals. However, this cost of meiosis arises only when there is a gene flow between sexual and asexual individuals (Barash 1976; Maynard Smith and Williams 1976). Mitochondrial phylogeny showed that sexual and asexual individuals of *T. tabaci* were in different phylogenetic groups from each other (Toda and Murai 2007), and the gene flow between sexual and asexual individuals is obscure. The microsatellite loci described in this study will clarify the gene flow.

**Table 1.**
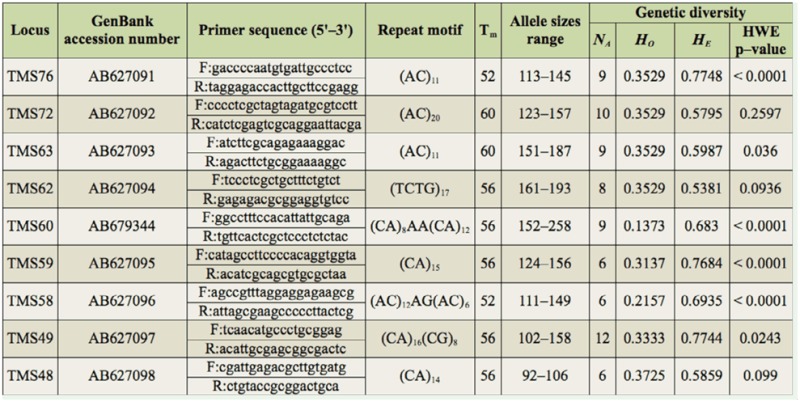
Primer sequence and diversity statistics for nine microsatellite loci isolated from *Thrips tabaci.* Shown are loci name, the GenBank accession numbers, the forward (F) and reverse (R) primer sequence, repeat motif of the sequenced clone, temperature at annealing (Tm), allele size range in base pairs, the number of alleles (NA), observed heterozygosity (HO), expected heterozygosity (HE) in the population (N = 51), and *p*-value associated with departure from Hardy-Weinberg Equilibrium (HWE) with Bonferroni correction.

**Table 2.**
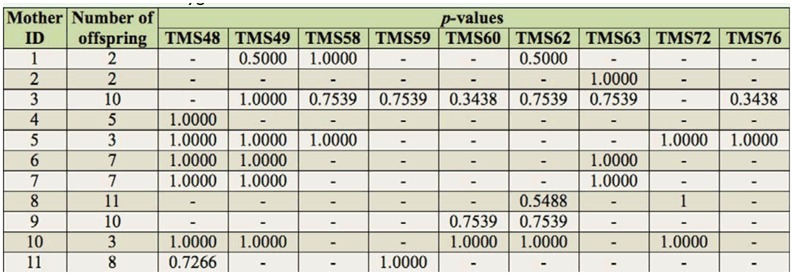
*p*-values of the binomial test to evaluate whether alleles on the nine microsatellite loci isolated from *Thrips tabaci* are inherited as Mendelian expectations. Only data of mothers bearing heterozygous locus are shown. A binomial test was conducted for each family and each locus to evaluate that the observed allele frequencies in offspring was different from 1:1. “-” indicates that the mother was homozygous at the locus.
